# Draft Genome Sequence and Polyhydroxyalkanoate Biosynthetic Potential of Jeongeupia naejangsanensis Type Strain DSM 24253

**DOI:** 10.1128/MRA.00167-21

**Published:** 2021-04-15

**Authors:** Paolo Turrini, Irene Artuso, Gabriele Andrea Lugli, Emanuela Frangipani, Marco Ventura, Paolo Visca

**Affiliations:** a Department of Science, Roma Tre University, Rome, Italy; b Laboratory of Probiogenomics, Department of Chemistry, Life Sciences, and Environmental Sustainability, University of Parma, Parma, Italy; c Department of Biomolecular Sciences, University of Urbino Carlo Bo, Urbino, Italy; University of Southern California

## Abstract

Jeongeupia naejangsanensis is a Gram-negative, cellulose-degrading betaproteobacterium. Here, we report the draft genome sequence of the type strain *J. naejangsanensis* DSM 24253 and identify the genes implicated in the biosynthesis of polyhydroxyalkanoate bioplastic polymers.

## ANNOUNCEMENT

**FIG 1:**
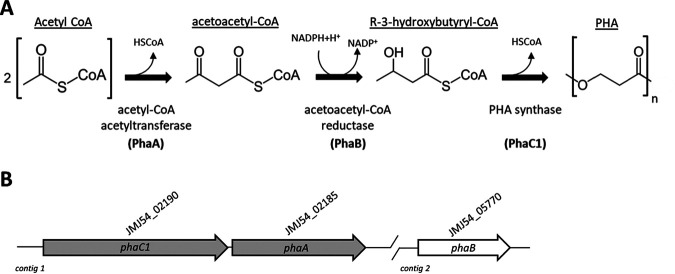
PHA biosynthetic pathway and genetic organization. (A) Biosynthesis of scl-PHAs from CoA takes place in three steps: the first reaction, catalyzed by acetyl-CoA acetyltransferase (encoded by *phaA*), consists of the condensation of two molecules of acetyl-CoA into acetoacetyl-CoA, which is reduced by acetoacetyl-CoA reductase (encoded by *phaB*) to R-3-hydroxybutyryl-CoA. Polymerization of R-3-hydroxybutyryl-CoA monomers is catalyzed by PHA synthase (encoded by *phaC1*). (B) Organization of the *phaC1A* operon and *phaB* gene in *J. naejangsanensis* DSM 24253^T^. Accessory genes (*phaC2PRZ*; see text) are scattered across the genome.

Jeongeupia naejangsanensis DSM 24253^T^ is a cellulose-degrading, aerobic, rod-shaped, motile β-proteobacterium belonging to the *Chromobacteriaceae* family, isolated from soil in South Korea (1). Two species of *Jeongeupia* have been formally described, *J. naejangsanensis* DSM 24253^T^ and *J. chitinilytica* DSM 28058^T^ (2). Recently, the whole genome of the unclassified *Jeongeupia* sp. strain USM3 was sequenced, and the genes implicated in the biosynthesis of polyhydroxyalkanoates (PHAs) were identified (3). PHAs are biodegradable thermoplastic polyesters and potential substitutes for synthetic plastics (4, 5). *Jeongeupia* sp. strain USM3 produces short-chain-length (scl) PHAs from acyl-coenzyme A (CoA) (Fig. 1A) that are used for nutrient storage (3). Given the biotechnological importance of PHAs, we determined the draft genome sequence of *J. naejangsanensis* DSM 24253^T^ and investigated the PHA biosynthesis genes in this type strain.

*J. naejangsanensis* DSM 24253^T^ was obtained from the DSMZ and aerobically grown at 28°C in Trypticase soy broth. DNA extraction was performed using a QIAamp DNA minikit (Qiagen). A genomic library was prepared for *J. naejangsanensis* using the TruSeq DNA PCR-free sample preparation kit (Illumina, Inc., San Diego, CA, USA). Genome sequencing was performed using a NextSeq 500 sequencing system, according to the supplier’s protocol (Illumina, UK), and the library samples were loaded into a midoutput kit v2.5 (300 cycles) (Illumina), producing 3,373,190 paired-end reads. The raw sequence reads were filtered and trimmed using the command-line software fastq-mcf (https://expressionanalysis.github.io/ea-utils/). Fastq files of Illumina paired-end reads (150 bp) were used as input in the MEGAnnotator pipeline for microbial genome assembly and annotation (6). This pipeline employed the program SPAdes v3.14.0 for *de novo* assembly of the genome sequence with the option “–careful” and a list of k-mer sizes (21,33,55,77,99,127) (7). The genome quality was evaluated with the program CheckM (8), estimating a genome completeness of 100% and 1.28% contamination. The contigs were submitted to the National Center for Biotechnology Information (NCBI) for the prediction of protein-encoding open reading frames (ORFs) and tRNA and rRNA genes using the NCBI Prokaryotic Genome Annotation Pipeline (PGAP) (9). All tools were run with default parameters unless otherwise specified.

The draft genome sequence of *J*. *naejangsanensis* DSM 24253^T^ is 3,910,756 bp long. It was assembled into 20 contigs with an *N*_50_ value of 300,395 bp, an average coverage of 237×, and a mean GC content of 63.63%. Genome annotation identified 3,748 ORFs, 52 tRNA genes, and 3 rRNA genes.

PHA-associated genes were identified in both the *J*. *naejangsanensis* DSM 24253^T^ and *J. chitinilytica* DSM 28058^T^ genomes, showing high similarity with those described in *Jeongeupia* sp. strain USM3 (Table 1). In these species, *phaC1* forms an operon with *phaA* (11), and they map apart from *phaB* (Table 1 and Fig. 1B). The phasin gene *phaP* is adjacent to *phaR*, which controls *phaP* expression and intracellular PHA accumulation (12). Two *phaZ* paralogs, encoding depolymerases involved in PHA mobilization (13), and the *phaC2* paralog, encoding an alternative PHA synthase (3), were also detected (Table 1).

**TABLE 1:** Relevant genes implicated in PHA metabolism in the *Jeongeupia* sp. strain USM3 genome with their homologs in *J*. *naejangsanensis* DSM 24253^T^ and *J. chitinilytica* DSM 28058^T^

Enzyme (gene)	Pfam family(ies)^*a*^	Locus tag in *Jeongeupia* sp. strain USM3	Locus tag in *J. naejangsanensis* DSM 24253^T^	% identity^*b*^ for:		Locus tag in *J. chitinilytica* DSM 28058^T^	% identity^*c*^ for:	
							BLASTn	BLASTp
				BLASTn	BLASTp			
Polyhydroxyalkanoate synthesis repressor (*phaR*)	PF05233, PF07879	BJP62_03065	JMJ54_01595	89	96	IE097_RS06190^*d*^	92
Phasin (*phaP*)	PF09361	BJP62_03070	JMJ54_01600	88	95	IE097_RS06185	89	96
Acetyl-CoA acetyltransferase (*phaA*)	PF00108, PF02803	BJP62_03585	JMJ54_02185	87	93	IE097_RS10880	88	95
Class I poly(R)-hydroxyalkanoic acid synthase (*phaC1*)	PF07167, PF00561	BJP62_03590	JMJ54_02190	83	87	IE097_RS10875	84	86
Acetyl-CoA acetyltransferase (*phaA*)	PF00108, PF02803	BJP62_03660	JMJ54_02270	94	98	IE097_RS10805	95	98
Acetoacetyl-CoA reductase (*phaB*)	PF13561	BJP62_04350	JMJ54_05770	83	86	IE097_RS03120	88	90
Alpha/beta hydrolase (*phaC2*)	PF07167	BJP62_08790	JMJ54_15395	85	84	IE097_RS15020	84	85
Poly(3-hydroxybutyrate) depolymerase (*phaZ*)	DUF3141	BJP62_12260	JMJ54_08070	88	86	IE097_RS09240^*d*^	90
Poly(3-hydroxybutyrate) depolymerase (*phaZ*)	PF10503	BJP62_12455	JMJ54_08295	91	92	IE097_RS09030	92	96

a Pfam family searches were performed in the Pfam database (10) at http://pfam.xfam.org.

b BLASTn/BLASTp analysis between *Jeongeupia* sp. strain USM3 PHA-associated genes/proteins and their corresponding homologs in *J. naejangsanensis* DSM 24253^T^.

c BLASTn/BLASTp analysis between *Jeongeupia* sp. strain USM3 PHA-associated genes/proteins and their corresponding homologs in *J. chitinilytica* DSM 28058^T^.

d BLASTp analysis was not performed since the corresponding *J. chitinilytica* DSM 28058^T^ gene was annotated as incomplete.

### Data availability.

This whole-genome shotgun project has been deposited at DDBJ/ENA/GenBank under accession number JAESND000000000. The version described in this paper is JAESND010000000. The raw sequencing reads are available at the Sequence Read Archive under accession number SRR13495155 and are associated with BioProject accession number PRJNA693669.
